# Antimicrobial stewardship: knowledge, perceptions, and factors associated with antibiotics misuse among consumer’s visiting the community pharmacies in a Nigeria Southwestern State

**DOI:** 10.1186/s40545-023-00629-x

**Published:** 2023-10-11

**Authors:** Wuraola Akande-Sholabi, Eunice Oyesiji

**Affiliations:** Department of Clinical Pharmacy and Pharmacy Administration, Faculty of Pharmacy, University of Ibadan, Ibadan, Nigeria

**Keywords:** Community pharmacy, Consumers, Antimicrobial resistance, Antibiotics misuse

## Abstract

**Background:**

In middle-income countries like Nigeria, the misuse of antibiotics by consumers is posing serious threats to public health. This is contributing to the alarming increase in antimicrobial resistance, which is reducing the effectiveness of antibiotics against common infections. This study therefore aimed to assess the knowledge, perceptions, and factors associated with antibiotics misuse among consumers visiting selected community pharmacies.

**Methods:**

This cross-sectional study conducted in Ibadan, Nigeria, aimed at determining factors influencing antibiotics misuse among consumers. The questionnaires were completed by 509 consumers. The analysis was done using SPSS version 26 and the results were presented using descriptive statistics. The associations between categorical variables were analysed using Pearson’s Chi-square with statistical significance set at *p* < 0.05.

**Results:**

Results showed that 95.9% of the consumers believed that antibiotics prevent bacterial growth, and 60.7% thought they treat all infections. However, 57.4% were unaware of antibiotic resistance, while only 14.7% had adequate knowledge about antibiotics. Most of the consumers, 72.5% had used antibiotics in the last 12 months and, amoxicillin 42.4% was the most commonly used with, malaria 38.9% as the primary condition for which antibiotics were used. Some of the significant factors influencing antibiotics misuse included delays in test reports (*p*-value = 0.007), the belief in antibiotics’ quick relief (*p*-value = 0.001), proximity of the pharmacy to their house or workplace (*p*-value = 0.028), amongst others.

**Conclusion:**

Most of the consumers had inadequate knowledge about rational antibiotic use which contributed to their misuse of antibiotics. Thus, targeted educational interventions are needed to improve knowledge and promote appropriate antibiotic use among consumers. Policies regulating the dispensing and selling of antibiotics with adequate counselling should be further enforced.

## Introduction

Antibiotics are substances that are used for inhibiting and treating infections caused by specific bacteria. They play an important role in treating bacterial infections, including life-threatening ones [1]. However, microorganisms are becoming less susceptible to antibiotics and by implication, infections are becoming difficult to treat because of antibiotics resistance [1]. Antibiotic resistance is a natural process [2], but the misuse and overuse of antibiotics have contributed to the constant surge in resistance, which is a global threat to human health [3].

Antibiotics resistance does not necessarily mean that the human body is resistant to antibiotics, instead, this phenomenon occurs when the growth of microbes is no longer inhibited or destroyed by antibiotics [1]. The continuous increase in the rate of antibiotic resistance is alarming and this poses imminent danger to the therapeutic effectiveness of antibiotics. As a result of this surge, resistant bacteria have become widespread [4]. This has also led to increased hospitalization rates, higher cost of health care, prolonged stay in hospital and faster mortality rates [5]. According to the United Nations (UN) ad hoc Interagency Coordinating Group (IACG), antibiotic-resistant infections currently cause approximately seven hundred thousand deaths yearly, and by 2050, this number could reach ten million annual deaths if urgent and effective measures are not taken to curb its spread [5]. It is therefore necessary to put in place strategies that will address and curb the dangers being posed to public health by antibiotic resistance.

The unethical antibiotics use by consumers has been favoured by community pharmacists dispensing these drugs without valid prescriptions [6]. In many middle-income countries, antibiotics are available over the counter [2, 7].

Many patients resort to antibiotic misuse due to factors such as lack of funds for doctor consultations, long wait times at hospitals, high costs of laboratory tests, and the unavailability of medical doctors [8]. Studies have shown that 75% of antibiotic requests and 60% of consultations with community pharmacists worldwide resulted in the sale of antibiotics without a valid prescription [8, 9].

Misuse of antibiotics such as failure to finish the antibiotic therapy, skipping of doses, reusing leftover medicines, and excessive use by consumers, have contributed to the widespread menace of antibiotic resistance globally [10]. This irrational use of antibiotics is posing danger to the health of the masses [8]. Based on a WHO report, about half of all medicines are prescribed, dispensed, or sold in ways that are inappropriate [11]. Unethical use of antibiotics is one of the topmost reasons for their reduced efficacy and effectiveness thus making common illnesses more difficult to treat [8, 11].

Although some research studies have reported the prevalence of antibiotic resistance in Nigeria, however, there is still inadequate information on the factors responsible for resistance in the country. Previous studies have identified cases of *Staphylococcus aureus* which is methicillin-resistant and *Escherichia coli* which is fluoroquinolone-resistant in Nigerian communities [12, 13]. Additionally, increased rates of antibiotic resistance have been observed among clinical specimens from a tertiary hospital in Nigeria [14]. However, only a small percentage of rational antibiotics use studies have been conducted in community pharmacies [15].

Given the global increase in antibiotic resistance, which is predominantly due to misuse of antibiotics. The aim of this study was to assess the knowledge, perceptions, and factors associated with antibiotics misuse among consumers visiting selected community pharmacies in Ibadan, Nigeria.

## Methods

### Study design and setting

This study was a questionnaire-guided cross-sectional survey that assessed the factors associated with antibiotic misuse among consumers visiting the community pharmacies in Ibadan. It was conducted in Ibadan, the capital city of Oyo State in Southwestern Nigeria. Oyo State is one of the 36 states in Nigeria, covering a landmass of 27 249 square kilometres. Ibadan has a population of about 3.6 million people, while the entire population of Oyo State is approximately 5.6 million people.

### Data collection instrument

The questionnaire used for the study was designed by the researchers after a thorough review of similar studies [8, 9, 16], as well as utilizing researchers’ proficiency. The designed questionnaire consisted of five sections. Section A focused on gathering socio-demographic data such as age, sex, marital status, educational level, ethnic group, occupation, residential area settlement, and the proximity of the pharmacy to their homes. Section B assessed the knowledge level of consumers about antibiotics and antibiotics resistance. In Section C, consumers were asked to provide their general perception regarding the use of antibiotics. Section D assessed the attitudes of consumers towards the use of antibiotics. Section E investigated the factors associated with antibiotic use among consumers.

### Inclusion and exclusion criteria

Eligible participants were consumers above 18 years that visited the community pharmacies during the study period. Those below the age of 18 and adults who were unwilling to participate in the research were excluded.

### Sample size determination

For the selection of community pharmacies in this study, simple random sampling technique was employed bearing in mind the centrality of the location and popularity in terms of patronage. At least one pharmacy was chosen from each of the 11 local governments in Ibadan. The managers of these selected pharmacies were then approached to provide an approximate count of the daily number of consumers they serve. By aggregating and averaging these estimates, an overall average of 1300 customers per month was determined. With a confidence level of 95% and a margin of error of 5%, the Yamane sample size formula [17] was utilized, resulting in a sample size of 314 for the consumer population. To account for a potential non-response of 10% and ensure an adequate sample size, the target sample population for consumers was adjusted to approximately 340. The number of consumers to be interviewed at each community pharmacy was proportionally allocated based on the estimated average number of customers per day.

### Sampling and data collection procedure

For the consumers, consecutive method of sampling was used. The study was conducted in selected community pharmacies in Ibadan, with at least one community pharmacy from each local government area. From each of the community pharmacies, the superintendent pharmacists were approached and the scope of the study was explained to them in order to seek their permission to carry out the study in their premises. In total, 24 community pharmacies gave their consent.

Subsequently, consumers visiting the pharmacies to purchase medication, refill their prescriptions, or seek health-related advice were approached. The study objectives were explained to them, and their informed consent was obtained from those who agreed to participate. Each consumer was interviewed using a questionnaire, which took not more than 20 min to complete. For respondents who did not understand English, a translated questionnaire was used, and their responses were back-translated. Consumers from each consented pharmacy were consecutively approached until the desired sample size was reached, with an average of 20 consumers interviewed per consented community pharmacy.

### Pre-test and content validation

The questionnaires underwent a thorough review by two clinical pharmacists to ensure the comprehensiveness and clarity. Additionally, a pre-test was conducted with consumers from the local community to assess the questionnaires’ ease of understanding. Based on the feedback received during the pre-test and the content validation process, slight adjustments were made to improve the questionnaires. The revised version of the questionnaires was then used to assess the knowledge, perception, attitude of consumers towards antibiotics uses and factors associated with antibiotics misuse among the consumers who visited the community pharmacies in Ibadan during the study period.

### Statistical analysis

After the questionnaires were collected, they were carefully checked for accuracy and assigned serial codes. Data entry, cleansing, and analysis were performed using the Statistical Package for Social Science (SPSS) version 26. Descriptive statistics, such as frequency counts, percentages, and means, were used to summarize and present the results and inferential statistics such as Pearson’s Chi-square was used to explore the association between statistical variables. The internal consistency/reliability of the 9-item knowledge and 7-item perception questions was determined using Cronbach alpha test, with a value of 0.68. For the consumers, their socio-demographic characteristics were summarized using descriptive statistics and presented in a table. Their knowledge about antibiotics and antibiotics resistance was assessed by assigning a score of “one” for each correct answer, while a score of “zero” was assigned for each incorrect answer. The overall scores for knowledge and perception questions were categorized as “good” or “poor” based on the respondents’ scores in each domain. The cut-off point for determining a “good” level of knowledge and perception was set at above 70%. Scores below the cut-off point were classified as “poor” knowledge and perception. The binary categorization for scoring the knowledge of the respondents was derived from Bloom’s cut-off criteria and other similar propositions [16, 18].

The questions used to assess their attitudes towards rational antibiotics use were analysed using descriptive statistics. Also, the factors influencing their use of antibiotics were identified using descriptive statistics where the 4-point Likert was converted to binary outcome of agree versus disagree such that the percentages of those who chose “strongly agree” or “agree” were considered as agree and the percentages of those who chose “strongly disagree” or “disagree” were considered as disagree. Pearson’s Chi-square test was used to evaluate the association between some socio-demographic characteristics and their knowledge, and also the association between their knowledge and factors associated with antibiotics use. The level of statistical significance was set at *p* < 0.05.

## Results

### Demographic characteristics of respondents

A total of 520 questionnaires were distributed among consumers visiting pharmacies in Ibadan to evaluate the factors contributing to inappropriate antibiotic dispensing. Among these, 509 questionnaires were adequately completed, resulting in 97.9% response rate. Out of the participants (258, 50.7%) were male, while 251 (49.3%) were female. Most of the participants were single (384, 75.4%), aged between 18–30 years (374, 73.5%), and resided in urban areas (400, 78.6%). A significant percentage of the respondents had attained tertiary education (424, 83.3%) (details in Table 1).

**Table 1:** Demographic characteristics of the respondents (*n* = 509)

Demographic factors	Variables	Frequency	Percentage
Gender	Male	258	50.7
	Female	251	49.3
Age group (years)	18–30	374	73.5
	31–40	88	17.3
	41–50	30	5.9
	≥ 51	17	3.3
Educational level	No formal education	2	0.4
	Primary education	6	1.2
	Secondary education	77	15.1
	Tertiary education	424	83.3
Occupation	Professional	96	18.9
	Public/civil servant	34	6.7
	Artisan	40	7.9
	Business personnel	99	19.4
	Farmer	7	1.4
	Student	169	33.2
	Unemployed	64	12.6
Marital Status	Single	384	75.4
	Married	120	23.6
	Divorced	2	0.4
	Separated	3	0.6
Residence	Rural	109	21.4
	Urban	400	78.6
Proximity of your house to the pharmacy	Very near	86	16.9
	Relatively near	123	24.2
	Not too far	175	34.4
	Far	125	24.6
Ethnicity	Yoruba	442	86.8
	Hausa	8	1.6
	Igbo	34	6.7
	Others	25	4.9

### Knowledge and perception of the respondents about antibiotics

A larger percentage of the consumers (488, 95.9%) expressed agreement that antibiotics have the ability to prevent or eliminate bacterial growth. Additionally, 309, 60.7% of them claimed that antibiotics can effectively treat all types of infections, including viral and fungal infections. Also, 247 (48.5%) respondents believed that antibiotics can be used to treat uncomplicated malaria. Interestingly, more than half of the respondents (292, 57.4%) admitted to not being aware of antibiotic resistance, while 342 (67.2%) acknowledged that antibiotic resistance refers to a phenomenon where bacteria lose their sensitivity to antibiotics. To assess their understanding of antibiotics and antibiotic resistance, they were presented with nine item questions, and (75, 14.7%) demonstrated adequate knowledge, whereas (434, 85.3%) had inadequate knowledge in this domain (details in Table 2).

**Table 2:** Knowledge of consumers about antibiotics (*n* = 509)

S/N	Questions	Yes *n* (%)	No *n* (%)	Unsure *n* (%)
1	An antibiotic is a drug that prevents or kills the growth of bacteria	488 (95.9)*	9 (1.8)	12 (2.4)
2	Antibiotics can be used to treat all infections including viral and fungal infections	309 (60.7)	133 (26.1)*	67 (13.2)
3	Antibiotics can be used in the treatment of cold, cough, sore throat and catarrh	346 (68)	91 (17.9)*	72 (14.1)
4	Antibiotics work best in the prevention of diarrhoea	199 (39.1)	125 (24.6)*	185 (36.3)
5	Antibiotics can help in the prevention of pregnancy after having unprotected sex	60 (17.1)	208 (59.4)*	82 (23.4)
6	Antibiotics can be used in the prevention of uncomplicated malaria	247 (48.5)	149 (29.3)*	113 (22.2)
7	Have you heard about Antibiotics Resistance?	292 (57.4)*	162 (31.8)	55 (10.8)
8	Misuse of antibiotics can increase the occurrence of antibiotics resistance	342 (67.2)*	31 (6.1)	136 (26.7)
9	Antibiotic resistance is a phenomenon where a bacterium loses its sensitivity to an antibiotic	354 (69.5)*	26 (5.1)	129 (25.3)

	Knowledge score	Frequency	Percentage	Remarks
	≥ 70	75	14.7	Adequate knowledge
	< 70	434	85.3	Inadequate knowledge

* Signifies the correct answer

Individual percent score = (individual score/9) × 100

Consumers’ perception of antibiotics was evaluated using a set of seven item questions, and the results showed that most of the respondents, (379, 74.5%), held a positive perception while only a few, (130, 25.5%), had a negative perception. Based on the appropriateness of purchasing antibiotics without a doctor's prescription, 433 respondents (85.1%) agreed that it is not suitable to do so whereas nearly half of them, (251, 49.3%), believed that antibiotic resistance is incapable of spreading to other individuals and causing death (details in Table 3).

**Table 3:** Perception of consumers on antibiotics (*n* = 509)

S/N	Questions	Yes *n* (%)	No *n* (%)	Unsure *n* (%)
1	Do you think antibiotics resistance can spread to other people and can cause death?	107 (21)*	251 (49.3)	151 (29.7)
2	Do you think antibiotics are safe for use when prescribed by a doctor?	477 (93.7)*	14 (2.8)	18 (3.5)
3	Do you think antibiotics might cause fatal side effects or allergic reactions?	345 (67.8)*	79 (15.5)	85 (16.7)
4	Do you think it is right to purchase antibiotics without a doctor’s prescription?	47 (9.2)	433(85.1)*	29 (5.7)
5	Do you think antibiotics are safe after their expiry dates?	22 (4.3)	450 (88.4)*	37 (7.3)
6	Do you think antibiotics are affected by storage conditions such as temperature, moisture or light?	379 (74.5)*	49 (9.6)	81 (15.9)
7	All antibiotics are safe within the recommended dose(s)	429 (84.3)*	30 (5.9)	50 (9.8)

	Perception score	Frequency	Percentage	Remarks
	≥ 70	379	74.5	Good perception
	< 70	130	25.5	Poor perception

* Signifies the correct answer

Individual percent score = (individual score/9) × 100

### Antibiotic usage by consumers, recommendations sources, and the use of prescription for its purchase

A significant majority of respondents (369, 72.5%) reported to have used antibiotics within the last 12 months. Among those respondents, slightly above half (297, 58.3%) acquired antibiotics through a prescription, while 144 (28.3%) obtained antibiotics without a prescription. The primary sources of antibiotic prescriptions or recommendations were pharmacists (38.6%) and doctors (37.1%). A smaller proportion of respondents (13.1%) engaged in self-medication, while 10.2% relied on recommendations from family and friends. In rare cases, 0.6% obtained antibiotics from a nurse, and 0.4% received them from a patent medicine personnel (details in Table 4).

**Table 4:** Antibiotic usage by consumers, recommendations sources, and the use of prescription for its purchase

Question	Variables	Frequency	Percentage
Estimated number of people who have used antibiotics in the last 12 months (*n* = 509)			
Have you used antibiotics in the last 12 months?	Yes	369	72.5
	No	140	27.5
Sources of antibiotic recommendations or prescriptions (*n* = 472)^a^			
Who prescribed or recommended the antibiotics you bought?	Doctor	175	37.1
	Pharmacist	182	38.6
	Yourself	62	13.1
	Friends and family	48	10.2
	Nurse	3	0.6
	Patent medicine personnel	2	0.4
Utilization of prescriptions for antibiotic purchases (*n* = 441)			
Did you buy the antibiotics without prescription?	Yes	144	28.3
	No	297	58.3

^a^Multiple responses

### Antibiotic utilization among consumers and associated medical conditions for the use in the last 12 months

Amoxicillin (42.4%) emerged as the most utilized antibiotic, followed by ampicillin (20.2%) and ciprofloxacin (19.4%), as indicated in Table 5. Conversely, ciprofloxacin + tinidazole, nitrofurantoin, and levofloxacin were infrequently used, with only one occurrence each. Among the medical conditions prompting antibiotic usage by consumers, malaria ranked highest (198, 38.9%), followed by cold/catarrh (101, 19.8%), sore throat (94, 18.5%), fever/headache (77, 15.1%), and urinary tract infection (76, 14.9%).

**Table 5:** Antibiotic utilization among consumers and associated medical conditions for the use in the last 12 months

	Variables	Frequency	Percentage
Antibiotics used by consumers in the last 12 months (*n* = 740)^a^			
The antibiotics	Amoxicillin	216	42.4
	Ampicillin	103	20.2
	Ciprofloxacin	99	19.4
	Tetracycline	75	14.7
	Metronidazole	70	13.8
	Amoxicillin + clavulanate	42	8.3
	Erythromycin	39	7.7
	Doxycycline	39	7.7
	Chloramphenicol	35	6.9
	Azithromycin	5	1.0
	Co-trimoxazole	3	0.6
	Cefuroxime	3	0.6
	Gentamicin	2	0.4
	Cephalexin	2	0.4
	Ofloxacin	2	0.4
	Streptomycin	2	0.4
	Nitrofurantoin	1	0.2
	Ciprofloxacin + tinidazole	1	0.2
	Levofloxacin	1	0.2
Medical conditions for antibiotic usage by consumers in the last 12 months (*n* = 569)^a^			
Medical conditions	Malaria	198	38.9
	Cold and catarrh	101	19.8
	Sore throat	94	18.5
	Fever/headache	77	15.1
	Cough	69	13.6
	Urinary tract infection	76	14.9
	Diarrhoea	50	9.8
	Skin infection	44	8.6
	GIT infection	40	7.9
	Ulcer	30	5.9
	STDs	21	4.1
	Ear infection	3	0.6
	Dental infection	2	0.4

^a^Multiple responses

### Duration of antibiotic use and reasons for discontinuation among consumers

Among the respondents, 264 (60.7%) reported using antibiotics for up to 5 days, while 87 (20%) used them for a duration of 7 days, and 46 (10.6%) used antibiotics for only 2 days. When asked about the reasons for discontinuing antibiotic use, completing the prescribed course was mentioned by 34.6%, stopped when they felt better by 35.8%, stopped based on the advice of a healthcare provider by 21.6%, and 19.8% stopped when their symptoms resolved. Other reasons included experiencing side effects (4.3%), perceiving the medication as ineffective (4.1%), and forgetting to take a dose (5.5%) (details in Table 6).

**Table 6:** Duration of antibiotic use and reasons for discontinuation among consumers

Question	Variables	Frequency	Percentage
Duration of antibiotic use among consumers in the last 12 months (*n* = 435)^a^			
For how long did you use the antibiotics?	1 day	2	0.4
	2 days	46	10.6
	≤ 5 days	264	60.7
	7 days	87	20
	More than 7 days	33	7.6
	Unsure	3	0.7
Reasons for the discontinuation of antibiotic use among consumers (*n* = 446)^a^			
When did you stop using the antibiotics?	When I finished the antibiotics	176	34.6
	When I felt better	182	35.8
	When the doctor or healthcare provider told me to stop	110	21.6
	When I didn't have the symptoms anymore	101	19.8
	When I experienced side effects	22	4.3
	When I noticed that the drug was not working	21	4.1
	When I forgot to take a dose	28	5.5

^a^Multiple responses

### Reported side effects of some antibiotics used by consumers

Some respondents experienced side effects from using antibiotics. Side effects reported included dry throat, rashes, diarrhoea, heartburn, and cough as a result of amoxicillin. Diarrhoea, stomachache, and side pain were reported as side effects of amoxicillin + clavulanic acid. Bitter mouth, dry mouth, and weakness were reported as side effects of ciprofloxacin. Additionally, other side effects were also reported (details in Table 7).

**Table 7:** Reported side effects of some antibiotics used by consumers (*n* = 22)

S/N	Reported side effects	Drugs	No of people	Percentage
1	Dry throat	Amoxicillin	1	4.6
2	Rashes	Amoxicillin	1	4.6
3	Diarrhoea	Amoxicillin	1	4.6
4	Heartburn	Amoxicillin	1	4.6
5	Cough	Amoxicillin	1	4.6
6	Headache	Amoxicillin	1	4.6
7	Weakness	Amoxicillin	1	4.6
8	Side pain	Amoxicillin + clavulanate	1	4.6
9	Diarrhoea and stomach ache	Amoxicillin + clavulanate	1	4.6
10	Body itching	Tetracycline	1	4.6
11	Excessive sleeping	Ampicillin	1	4.6
12	Dizziness	Ampicillin	2	9.0
13	Bitter mouth	Ciprofloxacin + tinidazole	2	9.0
14	Dry mouth	Ciprofloxacin	1	4.6
15	Weakness	Ciprofloxacin	1	4.6
16	Headache	Ciprofloxacin	1	4.6
17	Blood in sputum	Co-trimoxazole	1	4.6
18	Fever	Co-trimoxazole	1	4.6
19.	Headache	Azithromycin	1	4.6
20	Weakness and hunger	Erythromycin	1	4.6

### Factors associated with antibiotics use among consumers

When respondents were queried about their reasons for purchasing or using antibiotics, a variety of factors emerged. Specifically, (240, 47.1%) of participants cited the delay in obtaining test reports from the laboratory as a factor. Additionally, 385 (75.8%) agreed that antibiotics possess potent properties that offer quicker relief from symptoms. Moreover, 394 (77.4%) claimed that their usage was based on past prescriptions from doctors or other healthcare professionals, while 375 (73.7%) expressed comfort in using antibiotics after registering complaints with pharmacists. Furthermore, 243 (47.7%) acknowledged the convenience of nearby pharmacies in acquiring antibiotics and 164 (32.2%) of the respondents disclosed having a phobia for injections. It is noteworthy that 171 (33.5%) expressed a preference for antibiotics over expensive consultation fees, and 70 (13.8%) admitted to occasionally combining antibiotics with herbal preparations (details in Table 8).

**Table 8:** Factors associated with antibiotics use among consumers (*n* = 509)

	Why do you use or buy antibiotics?	Strongly agree *n* (%)	Agree *n* (%)	Neutral *n* (%)	Disagree *n* (%)	Strongly disagree *n* (%)
1	Laboratory reports take longer before they are ready, so I opt for antibiotics	80 (15.7)	160 (31.4)	102 (20)	97 (19.1)	70 (13.8)
2	Antibiotics are powerful drugs that relieve symptoms faster	157 (30.8)	228 (45)	92 (18.1)	24 (4.7)	7(1.4)
3	I use antibiotics because it was once prescribed by my doctor/ healthcare provider	200 (39.3)	194 (38.1)	56 (11.0)	38 (7.5)	21 (4.1)
4	I do not like visiting the hospital because it is time wasting	72 (14.1)	77 (15.1)	102 (20)	138 (27.1)	120 (23.6)
5	Rather than paying heavily for a consultation with a doctor, I prefer to use antibiotics	72 (14.1)	99 (19.4)	86 (16.9)	131 (25.7)	121 (23.8)
6	As a result of my busy schedule, I go for antibiotics when I feel unwell instead of seeing a medical doctor	62 (12.2)	97 (19.1)	105(20.6)	134 (26.3)	111(21.8)
7	The pharmacy’s closeness to my house or workplace, makes it easier for me to buy antibiotics when I am sick	106 (20.8)	137 (26.9)	92 (18.1)	100 (19.6)	74 (14.5)
8	I use leftover antibiotics when I do not have enough money to afford a new one	64 (12.6)	83 (16.3)	76 (14.9)	139 (27.3)	147 (28.9)
9	I have phobia for injections which are mostly administered in the hospital, so when I am ill, I opt for antibiotics instead	76 (14.9)	88 (17.3)	98 (19.3)	115 (22.6)	132 (25.9)
10	If I skip one or two doses of my drug, I compensate that by using double doses	32 (6.3)	32 (6.3)	65 (12.8)	129 (25.3)	251 (49.3)
11	I sometimes mix antibiotics with other herbal preparations for effectiveness	32 (6.3)	38 (7.5)	73 (14.3)	115 (22.6)	251(49.3)
12	I buy antibiotics to use since they are readily available at the pharmacy without prescription	57 (11.2)	97 (19.1)	92 (18.1)	124 (24.4)	138 (27.3)
13	If my family member or friend is ill, I can give him/her from my antibiotics especially if we have similar symptoms	47 (9.2)	112 (22.0)	68 (13.4)	118 (23.2)	164 (32.2)
14	I do not need to visit a doctor for a prescription if I know the antibiotics to use when I am sick	65 (12.8)	110 (21.6)	88 (17.3)	122 (24.0)	124 (24.4)
15	I use antibiotics when I feel symptoms of sickness	58 (11.4)	94 (18.5)	97 (19.1)	136 (26.7)	124 (24.4)
16	If it is a minor illness, I can take the initiative to use antibiotics	48 (9.4)	109 (21.4)	114 (22.4)	130 (25.5)	108 (21.2)
17	After lodging my complaint to the pharmacist, I find it comfortable to use the antibiotics given to me	202 (39.7)	173 (34.0)	68 (13.4)	36 (7.1)	30 (5.9)
18	Pressures from families and friends usually make me use antibiotics even if I do not want to, especially when I am sick	66 (13.0)	67 (13.2)	84 (16.5)	130 (25.5)	162 (31.8)
19	I buy antibiotics in bulk, in order to have them at home in case of any emergency	43 (8.4)	59 (11.6)	66 (13.0)	140 (27.5)	201 (39.5)
20	I do not trust physicians and nurses, so I buy the antibiotics for myself without their advice	24 (4.7)	20 (3.9)	53 (10.4)	137 (26.9)	275 (54.0)

### Association between knowledge and some socio-demographic characteristics of consumers

Table 9 shows the association between knowledge of the consumers and their various socio-demographic characteristics. The table examines the variables of gender, age group, educational level, occupation, and proximity of residence to the pharmacy. The focus is on identifying any statistically significant associations with poor knowledge (score < 70%) and good knowledge (score ≥ 70%).

**Table 9:** Association between knowledge and some socio-demographic characteristics of consumers (*n* = 509)

	Demographic factors	Variables	Poor knowledge (score < 70%) *n* (%)	Good knowledge (score ≥ 70%) *n* (%)
1	Gender	Male	218 (84.5)	40 (15.5)
		Female	216 (86.1)	35 (13.9)
*X*^2^ = 0.620, *p*-value = 0.246				
2	Age group	18–30	315 (84.2)	59 (15.8)
		31–40	80 (90.9)	8 (9.1)
		41–50	26 (86.7)	4 (13.3)
		≥ 51	13 (76.5)	4 (23.5)
*X*^2^ = 3.647, *p*-value = 0.302				
3	Educational level	No formal education	1 (50)	1 (50)
		Primary education	6 (100)	0 (0)
		Secondary education	68 (88.3)	9 (11.7)
		Tertiary Education	359 (84.7)	65 (15.3)
*X*^2^ = 3.705, *p*-value = 0.295				
4	Occupation	Professional	75 (78.1)	21 (21.9)
		Public/civil servant	28 (82.4)	
		Artisan	39 (97.5)	1 (2.5)
		Business Personnel	89 (89.9)	10 (10.1)
		Farmer	7 (100)	0 (0)
		Student	137 (81.1)	32 (18.9)
		Unemployed	59 (92.2)	5 (7.8)
*X*^2^ = 16.607, *p*-value = 0.011*				
5	Proximity of residence to the pharmacy	Very near	67 (77.9)	19 (22.1)
		Relatively near	102 (82.9)	21 (17.1)
		Not too far	154 (88)	21 (12)
		Far	111 (88.8)	14 (11.2)
*X*^2^ = 6.526, *p*-value = 0.089				

* Statistically significant at *p* < 0.05 *X*^2^ = Chi-square

It was observed that there was no statistically significant association between gender, age groups, educational level, and proximity of residence with knowledge level (*p*-value = 0.246, *p*-value = 0.302, *p*-value = 0.295, and *p*-value = 0.089).

However, when examining the occupation variable, a statistically significant association was observed. The occupation of consumers was found to have a significant impact on their knowledge level (*p*-value = 0.011). Specifically, professionals, public/civil servants, artisans, business personnel, and students had higher levels of good knowledge, while unemployed individuals had a higher percentage of poor knowledge.

### Association between knowledge and factors associated with antibiotics use among consumers

Table 10 examines the association between knowledge levels and various factors influencing the purchase or use of antibiotics among consumers. The results revealed several statistically significant associations with knowledge level. Individuals with poor knowledge were more likely to indicate that laboratory reports took longer before they were ready (*p*-value = 0.007), believed in the fast symptom relief of powerful antibiotics (*p*-value = 0.001), used antibiotics based on past prescriptions (*p*-value = 0.000), considered the proximity of the pharmacy to their house or workplace as a determining factor (*p*-value = 0.028), used antibiotics when feeling sick (*p*-value = 0.001), took the initiative to use antibiotics for minor illnesses (*p*-value = 0.000), and expressed a lack of trust in physicians and nurses leading to self-purchasing of antibiotics (*p*-value = 0.039). Conversely, individuals with good knowledge were less likely to hold these beliefs or engage in such behaviours.

**Table 10:** Association between knowledge and factors associated with antibiotics use among consumers (*n* = 509)

	Why do you buy/use antibiotics?	Responses	Poor knowledge (Score < 70%) *n* (%)	Good knowledge (Score ≥ 70%) *n* (%)
1	Laboratory reports take longer before they are ready, so I opt for antibiotics	Strongly agree	73 (91.3)	7 (8.7)
		Agree	136 (85)	24 (15)
		Neutral	93 (91.2)	9 (8.8)
		Disagree	81 (83.5)	16 (16.5)
		Strongly Disagree	51 (72.9)	19 (27.1)
*X*^2^ = 13.944, *p*-value = 0.007*				
2	Antibiotics are powerful drugs that relieve symptoms faster	Strongly agree	141 (89.8)	16 (10.2)
		Agree	197 (86)	32 (14)
		Neutral	75 (81.5)	17 (18.5)
		Disagree	14 (58.3)	10 (41.7)
		Strongly disagree	7 (100)	0 (0)
*X*^2^ = 18.777, *p*-value = 0.001*				
3	I use antibiotics because it was once prescribed by my doctor/ healthcare provider	Strongly agree	173 (86.5)	27 (13.5)
		Agree	172 (88.7)	22 (11.2)
		Neutral	49 (87.5)	7 (12.5)
		Disagree	29 (76.3)	9 (23.7)
		Strongly disagree	11 (52.4)	10 (47.6)
*X*^2^ = 22.742, *p*-value = 0.000*				
4	The pharmacy’s closeness to my house or workplace, makes it easier for me to buy antibiotics when I am sick	Strongly agree	98 (92.5)	8 (7.5)
		Agree	120 (87.6)	17 (12.4)
		Neutral	79 (85.9)	13 (14.1)
		Disagree	79 (79)	21 (21)
		Strongly disagree	58 (78.4)	16 (21.6)
*X*^2^ = 10.893, *p*-value = 0.028*				
5	I use antibiotics when I feel symptoms of sickness	Strongly agree	54 (93.1)	4 (6.9)
		Agree	85 (90.4)	9 (9.6)
		Neutral	90 (92.8)	7 (7.2)
		Disagree	108 (79.4)	28 (20.6)
		Strongly disagree	97 (78.2)	27 (21.8)
*X*^2^ = 17.792, *p*-value = 0.001*				
6	If it is a minor illness, I can take the initiative to use antibiotics	Strongly agree	45 (93.8)	3 (6.2)
		Agree	100 (91.7)	9 (8.3)
		Neutral	103 (90.4)	11 (9.6)
		Disagree	106 (81.5)	24 (18.5)
		Strongly disagree	80 (74.1)	28 (25.9)
*X*^2^ = 20.941, *p*-value = 0.000*				
7	I do not trust physicians and nurses, so I buy the antibiotics for myself without their advice	Strongly agree	24 (100)	0 (0)
		Agree	17 (85)	3 (15)
		Neutral	49 (92.5)	4 (7.5)
		Disagree	120 (87.6)	17 (12.4)
		Strongly disagree	224 (81.5)	51 (18.5)
*X*^2^ = 10.096, *p*-value = 0.039*				

* Statistically significant at *p* < 0.05 *X*^2^ = Chi-square

## Discussion

Findings from this study underscore the irrational use of antibiotics by consumers and a significant gap in their knowledge about rational antibiotics use even though a larger percentage of them have tertiary education. These results differ from studies conducted in Tanzania, where individuals with higher education levels demonstrated better understanding of antibiotic use [8, 18–20].

More than half of the respondents were of the belief that antibiotics could treat all infections, including viral and fungal infections, and held misconceptions such as the belief that antibiotics can prevent diarrhoea or act as a preventive measure after unprotected sex. These perceptions align with previous research highlighting similar misconceptions which stem from limited knowledge, societal influences, and a desire for quick health solutions [21–26]. However, it is necessary to address these beliefs due to their potential consequences, such as the misuse and overuse of antibiotics, which are contributory factors to the global challenge of antibiotic resistance.

One of the threats posed by antibiotic resistance is a high mortality rate. Unfortunately, about half of the respondents in this study believed that antibiotic resistance cannot spread to other people and cannot cause death. This wide knowledge gap is a contributory factor to the irrational use of antibiotics. While most of them understood that antibiotic resistance occurs when bacteria are no longer sensitive to antibiotics and acknowledged that misuse can increase resistance, some respondents claimed to have never heard about antibiotic resistance. This lack of awareness aligns with similar findings in a study by Khan et al*.* in 2022 where consumers had poor knowledge of antibiotic use and limited understanding of antibiotic resistance [27].

The top five antibiotics reportedly used by consumers in this study in the last 12 months were amoxicillin, ampicillin, ciprofloxacin, tetracycline, and metronidazole. The widespread use of amoxicillin can be attributed to its affordability and availability as an over-the-counter medicine in pharmacies which has enabled individuals to self-medicate with it for common infections. Also, the frequent use of tetracycline and metronidazole suggests their utilization for the prevention or treatment of diarrhoea, as about a third of respondents believed antibiotics are most effective for preventing diarrhoea, which is also one of the top seven ailments for which consumers used antibiotics in this study. Similar misuse of amoxicillin has been reported in Indonesia, while Ampiclox (ampicillin–cloxacillin) was found to be the most misused antibiotic among consumers in Lagos and Ilorin, Nigeria [28–30].

Malaria emerged as the most common reason for antibiotic use among consumers, despite the fact that it is caused by protozoa (*Plasmodium*) and not bacteria. Alarmingly, close to half of the consumers believed that antibiotics are used to treat uncomplicated malaria. Well, malaria which is a life-threatening disease with high prevalence in tropical regions is best treated with antimalarial medicines, however, some antibiotics like macrolides and tetracycline have proven to be effective in its treatment [16, 31]. Nonetheless, the irrational use of antibiotics for uncomplicated malaria is setting them up for resistance to bacteria. This is in contrast with a study done by Gabriel et al*.* [32] where 60.5% of respondents correctly believed that malaria is not treatable with antibiotics whereas, in the Northern zone of Tanzania, 68.4% of the participants agreed that antibiotics can be used in the treatment of malaria [19].

Cold, cough, sore throat and catarrh were among the top five ailments for which consumers used antibiotics. However, these ailments are primarily viral and self-limiting, and do not require antibiotics for treatment [33, 34]. This misuse of antibiotics aligns with the knowledge gap identified in this study, where 68% of consumers were of the opinion that antibiotics could be used to treat these illnesses. Some studies conducted in different countries, including Britain, South Korea, Mongolia, and Qatar, have identified poor consumer knowledge about the effectiveness of antibiotics for coughs and colds. A higher percentage of respondents in these studies claimed ignorance about the fact that antibiotics are not effective against most coughs and colds. Such widespread misuse of antibiotics for viral infections contributes to the spread of antibiotic resistance [35–39]. Therefore, it is necessary to educate the public on the differences between viral and bacterial infections so as to combat antibiotic resistance effectively.

A concerning trend was observed in this study, with nearly half of the consumers obtaining antibiotics with prescriptions or recommendations from pharmacists, while over a quarter acquired antibiotics without any prescription. The roles of pharmacists in the healthcare team are distinct from those of the physicians. However, it is unfortunate that many pharmacists now prescribe antibiotics, which are Prescription Only Medicines, to patients. This practice has contributed to the proliferation of antibiotic resistance due to improper disease management. Several studies have reported the involvement of community pharmacists in promoting self-medication with antibiotics by dispensing them without prescription [40–42]. Moreover, pharmacists have been identified as key players in dispensing incomplete doses of antibiotics to boost sales [43]. These practices call for proper patient education on rational antibiotic use and stricter regulations on the prescription and dispensing of antibiotics.

In addition to obtaining antibiotics from pharmacies, some consumers engaged in self-medication or received recommendations from friends and family. This practice could explain why approximately 10.6% of the respondents used antibiotics for only two days, despite antibiotic therapy typically requiring longer durations. This is consistent with the study done in Ilorin, Nigeria, where 23% of respondents took antibiotics for only 2 days [30]. Incomplete antibiotic dosage regimens contribute to the increase in antibiotic resistance and the transmission of resistant bacteria. Incomplete antibiotic dosage regimens can lead to the emergence of antibiotic resistance in the body due to sub-inhibitory concentrations of the antibiotics. Moreover, incomplete treatment can result in recurring infections that become difficult to treat [44, 45]. Thus, patient education on rational antibiotic use is crucial to address this issue. Regarding antibiotic discontinuation, most respondents reported stopping antibiotic use when they felt better or when their symptoms disappeared. Some individuals discontinued use due to the side effects of the drugs. Some of the reported side effects included dry throat, rashes, diarrhoea, heartburn, and cough which were associated with amoxicillin. Ciprofloxacin was associated with side effects like weakness and a bitter and dry mouth. These common side effects align with those reported in a study carried out in North Central Nigeria [30]. Although the severity of these side effects was not investigated in this study, however, they could contribute to consumers prematurely stopping antibiotic regimens. The premature discontinuation of antibiotic therapy can lead to incomplete eradication of bacteria, allowing the surviving bacteria to develop resistance to the antibiotic. It is necessary for consumers to finish the entire course of antibiotics as prescribed by healthcare professionals.

Additionally, this study revealed that consumers engage in misuse of antibiotics, such as self-medication, using incomplete therapy, and using antibiotics for conditions not caused by bacteria. Interestingly, despite a larger percentage of respondents having tertiary education, a significant knowledge gap was observed among consumers visiting pharmacies. This emphasizes the need for increased public enlightenment and awareness programmes, which can be effectively implemented by pharmacists within the pharmacy setting.

To further understand the factors contributing to antibiotics misuse by consumers, they were asked about their reasons for buying or using antibiotics, and several responses were recorded so as to shed more light on the factors responsible for antibiotic misuse.

A considerable number of respondents displayed a strong inclination to choose antibiotics due to the delay in receiving laboratory reports. This suggests that the waiting period for test results may drive individuals to resort to self-medication with antibiotics [46]. Another notable factor is the belief that antibiotics are powerful drugs that provide rapid symptom relief. This belief, held by a significant portion of the respondents, may stem from a lack of understanding about the right use of antibiotics and their specific indications. This misconception can contribute to the misuse of antibiotics even in the absence of bacterial infection [2]. Addressing this misconception is crucial in combating antibiotic resistance.

The influence of healthcare providers is evident, as some individuals reported using antibiotics based on past prescriptions from doctors or other healthcare providers. This highlights the importance of responsible prescribing practices and patient education. Healthcare professionals play a crucial role in communicating the rationale behind antibiotic prescriptions, ensuring that patients understand the necessity and appropriate duration of treatment [47].

Additionally, nearly half of the respondents mentioned the convenience of having a nearby pharmacy as a factor that facilitated their access to antibiotics. This finding suggests that the accessibility and proximity of pharmacies influence consumer choices, potentially enabling easy access to antibiotics. It underscores the need for stricter regulations and the enforcement of prescription-only policies to prevent the inappropriate sale of antibiotics [48].

A significant proportion indicated that they had a phobia of injections. This fear may lead individuals to prefer antibiotic use over receiving injectable medications, highlighting a personal factor influencing their decision-making process. Also, approximately one-third of respondents expressed a preference for using antibiotics rather than paying hefty consultation fees. This financial consideration reflects a tendency to prioritize cost-effectiveness over seeking professional medical advice, potentially leading to inappropriate antibiotic use [49].

Furthermore, the belief that minor illnesses and its symptoms can be managed by taking the initiative to use antibiotics is another significant factor contributing to their misuse. This is similar to the research conducted in the Northern zone of Tanzania where over half of the participants believed that antibiotics can be used to treat minor illnesses [19]. This self-perception of the ability to determine the necessity of antibiotics may stem from a lack of understanding about the risks associated with inappropriate use and the potential development of antibiotic resistance. Thus, enhancing public knowledge on the proper use of antibiotics and the importance of seeking professional advice for proper diagnosis is crucial in addressing this issue [39].

Moreover, a smaller proportion (13.8%) admitted to occasionally mixing antibiotics with other herbal preparations. This practice raises concerns about the potential interaction between antibiotics and herbal remedies, emphasizing the need for proper guidance from healthcare professionals.

A considerable number of respondents claimed that after reporting their complaints to pharmacists, they found it comfortable to use the antibiotics provided. This suggests that the reassurance and recommendations received from pharmacists are crucial in the decision-making process regarding antibiotic use [50]. However, a small percentage of respondents demonstrated a notable distrust in physicians and nurses, leading them to purchase antibiotics without professional advice. This lack of trust in healthcare providers contributes to self-medication practices and hinders appropriate antibiotic use. Thus, strengthening the patient–provider relationship and promoting trust through effective communication can discourage self-medication and empower patients to make informed decisions [51].

To combat antibiotic misuse, a comprehensive and collaborative approach involving healthcare professionals, policymakers, technology developers, and the public is crucial. Healthcare professionals should focus on patient education and communication to improve understanding of proper antibiotic use [51]. Policy interventions, such as stricter enforcement of prescription-only regulations, are necessary to regulate antibiotic availability and discourage self-medication [49]. Technological advancements, including digital health tools, can support patient adherence and informed decision-making [52]. Targeted public awareness campaigns tailored to specific populations can address misconceptions and promote responsible antibiotic use [47]. By implementing these strategies, antibiotic stewardship can be enhanced and the risks associated with antibiotic resistance can be curbed so as to safeguard the efficacy of these life-saving medications for the future.

## Limitations of the study

The nature of this research gives room for both response and recall bias. It is possible that respondents may have provided answers that were influenced by their current circumstances or personal biases. Moreover, the study solely relied on data from community pharmacies and did not capture responses from individuals who obtained their antibiotics from other sources, such as hospitals or online platforms. Furthermore, the translated questionnaire was not revalidated. Hence, the findings from this research may not be fully representative of the broader population, and caution on generalization.

Despite these limitations, this study has provided valuable insights into the knowledge, perception, and attitude of consumers towards rational antibiotic use and the factors influencing their misuse of antibiotics. By addressing these factors and paying close attention to the findings, it is possible to enhance patient care and curb the spread of antibiotic resistance in Nigeria.

## Conclusion

This study highlights important findings about the knowledge, perception, and attitudes of consumers in relation to the use of antibiotics. Most of the consumers had poor knowledge about antibiotics, which is a contributory factor to their misuse of these medications. In addressing this, it is paramount to increase awareness campaigns about the dangers associated with antibiotics misuse. Also, educating them on proper antibiotic use is equally important so as to ensure that they understand the right indications, dosages, and duration of treatment. By addressing these gaps in knowledge, the spread of antibiotic resistance can be effectively curbed. Policies regulating the dispensing and selling of antibiotics with adequate counselling should be further enforced.

## Data Availability

The datasets used and/or analysed during the current study are available from the corresponding author on reasonable request.

## Abbreviation

WHO: World Health Organization
